# An Attempt to Construct an Antiseptic Mouthwash

**Published:** 1888-04

**Authors:** W. D. Miller

**Affiliations:** Berlin


### AN ATTEMPT TO CONSTRUCT AN ANTISEPTIC MOUTHWASH.

BY W. D. MILLER, BERLIN.

When at the beginning of the present decade, through the most exact methods of bacteriological investigation now in use, the true (parasitic) cause of one disease after another was brought to light, we had many reasons to hope that the helpless position of medicine in the presence of the severest infectious diseases was soon to be changed. As yet, however, our expectations have not been realized. With the exception of the still somewhat doubtful triumphs of Pasteur over anthrax and hydrophobia, very little advantage whatever has resulted to therapeutics from the eminent bacteriological discoveries of the last ten years. Consumption, cholera, typhus, diphtheria, syphilis, have not become less terrible through the discovery of the specific micro-organisms of these disorders. Diseases which come under the treatment of the dentist form no exception to this statement.

The fact that decay of the teeth is of parasitic origin having been once established, the thought suggests itself that we ought to be able, by means of properly chosen antiseptic materials, not only to arrest decay, but to prevent its appearance. This is, indeed, the avowed object of the very many antiseptic mouthwashes now in the market. As a matter of fact, however, there is no evidence that anything whatever has as yet been accomplished in the prophylactic treatment of the teeth through the use of antiseptic mouthwashes, and it is evident that any one who would discover some means by which the often fatal ravages caused by decay of the teeth might be held in check, would thereby confer a great boon on humanity.

Not in the hope of accomplishing this, but of contributing something towards its accomplishment, the experiments were made which form the subject of this paper.

The object of the experiments has been to find some substance or some mixture which might be used with impunity in the human mouth, and at the same time be sufficiently strong to effect an approximately thorough sterilization of the same. None of the many mouthwashes with which I am acquainted, unless we except Listerine, makes even an approach to accomplishing this, the majority of them having at most only a slightly astringent action and an agreeable odor and taste. In fact, there is some ground for the assertion that mouthwashes have done more harm than good.

People who have not the patience or energy to give their teeth a thorough cleaning, quiet their consciences by rinsing their mouths with some "antiseptic" mouthwash, in the belief that they are thereby doing all that is really necessary to insure the conservation of their teeth, whereas, in fact, the chief agent for the conservation of the teeth is the toothbrush; without it the strongest antiseptics will accomplish very little, and any one who puts aside the toothbrush for a mouthwash does it to his own harm.

A few experiments having the present object in view were made about four years ago, and published in the INDEPENDENT PRACTITIONER for August, 1884.

During the present winter I have extended my experiments somewhat, and added a few new substances to the materials experimented upon. The chief of these were salol, aseptin and acetate of aluminium.

*Salol* was first recommended by Sahli as a mouthwash. A teaspoonful of the concentrated alcoholic solution added to half a glass of water gives an emulsion, the particles of which are supposed to be deposited in the fissures and cavities of the teeth, where they exert a permanent antiseptic action. Sahli saw good results from its use in a case of soor, the growth disappearing after a very few applications. Salol is a very pleasant remedy, but unfortunately I have found that it is almost useless as a mouthwash, solutions much stronger than the one given above producing almost no action whatever in either of the series of experiments described below.

Aseptin is an antiseptic of low power, compared to thymol, carbolic acid, etc., but it has a decided advantage over them, in that it may be used in much more concentrated solutions. Fifty per cent.

solutions (in which strength it may be used in the mouth) have a considerable antiseptic power, and may take the place of water in preparing mouthwashes, as suggested in solution No. 6. It does not, however, in combination with other antiseptics, increase their power in the expected degree. The same appears, however, to be true of all antiseptics which I have examined in regard to this question. If we combine two antiseptics, each of which has a certain action in one minute, it does not follow that the mixture would exert the same action in half a minute, or double the action in one minute.

Acetate of aluminium is an old medicament, still adhered to by many physicians; it combines considerable antiseptic power with a very strong astringent action. The strongest solution which can be used in the human mouth had in some cases a marked action, but on the whole, not strong enough to encourage me in its use.

My aim in the experiments to be ascribed was to combine a number of antiseptics in such a manner as to produce the greatest possible antiseptic action, with the least possible action on the mucous membrane. The fact that those substances which have a marked deleterious action upon the protoplasm of micro-organisms, act at the same time either as powerful local irritants upon the animal tissue to which they are employed, or as systemic poisons, or both together, has proved to be one of the greatest, or indeed the greatest, hindrance in the use of antiseptics in counteracting infectious diseases. The same difficulty is encountered in a high degree in the attempt to sterilize the human mouth. The sensitiveness and delicacy of the mucous membrane of the human mouth excludes the use of all escharotic materials except in very dilute solutions; other materials of great value, such as bichloride of murcury, we hesitate to use through fear that the small quantities taken up by the mucous membrane or swallowed may produce mercurial poisoning.

A third class of antiseptics is excluded because they have a solvent action upon the teeth, and still others because they have a disagreeable smell or taste.

For these various reasons, the task of finding a mouthwash for daily use which has a pronounced devitalizing action upon the bacteria of the oral cavity is one of exceeding great difficulty, and one which unfortunately will probably never be thoroughly accomplished.

My experiments were made on the following mixtures:

| | | |
|---|---|---|
| (1.) | Bichloride of mercury | 0.025 |
| | Water | 50.00 |
| (2.) | Water | 50.00 |
| | Alcohol | 5.00 |
| | Tinct. eucalypt | 0.75 |
| | Benzoic acid | 0.15 |
| | Thymol | 0.0125 |
| (3.) | Listerine | 25.0 |
| | Water | 25.0 |
| (4.) | Aseptin | 25.00 |
| | Water | 25.00 |
| | Alcohol | 5.00 |
| | Tinct. eucalypt | 0.75 |
| | Benzoic acid | 0.15 |
| | Thymol | 0.0125 |
| (5.) | Water | 50.00 |
| | Alcohol | 5.00 |
| | Tinct. eucalypt | 0.75 |
| | Benzoic acid | 0.15 |
| | Thymol | 0.0125 |
| | Bichloride of mercury | 0.025 |
| (6.) | Water | 25.00 |
| | Aseptin | 25.00 |
| | Salol | 5.00 |
| | Alcohol | 5.00 |
| | Acetate of aluminium | 1.50 |
| | Benzoic acid | 0.15 |
| | Thymol | 0.0125 |
| | Tinct. eucalypt | 0.75 |

*These mixtures are not mouthwashes,* but they might serve as bases for mouthwashes, as indicated at the end of this article.

The alcohol was added only as a solvent, not because of its antiseptic powers.

With these mixtures a series of experiments were made. The first series was carried out in the manner described in the INDEPENDENT PRACTITIONER for August, 1884.

In this series over 1,500 inoculations were made, and the results obtained were found to be somewhat at variance with those published previously, in so far as longer time was required by the different solutions to devitalize the micro-organisms acted upon, than is given in the table published in the INDEPENDENT PRACTITIONER.

This difference may, however, be readily accounted for by the fact that I, this time, made use of a different bacterium, which, without any doubt, has a greater power of resistance than those before experimented upon.

As a mouthwash, we need above all a solution which acts *quickly*, and which does not simply prevent the development of micro-organisms while it is acting, but which devitalizes them.

There are agents which, even in very dilute form, if applied constantly have a powerful antiseptic action, inasmuch as they prevent the development of such micro-organisms as may be present without, however, devitalizing them; such agents are of no more value as antiseptics in the treatment of the oral cavity than an equal amount of distilled water. It is seldom that any one in rinsing his mouth will retain the wash longer than one minute, and an antiseptic mouthwash, to be efficient, should be able to devitalize the micro-organisms with which it comes in contact within this short time.

Solution No. 5 accomplishes this for nearly, if not for all, micro-organisms in the vegetative form. A solution which devitalizes spores in one minute is out of the question, and, in fact, is not at all necessary, since the conditions which lead to the formation of spores do not exist in the mouth, where we find almost exclusively the vegetative forms.

This solution (No. 5) has a decided action in one-fourth to one-half minute; in one minute the sterilization is nearly or quite complete.

Next to this came the solutions Nos. 6, 4 and 2, in close order; the addition of aseptin and acetate of aluminium, both of which, but particularly the former, are antiseptics of considerable strength, did not produce the hoped-for increase in the action of the solution.

The addition of salol had, as I anticipated, no effect whatever. These solutions produced a decided diminution in the number of colonies in a half minute; a complete sterilization usually required two minutes, sometimes even longer.

Nearly as strong as these solutions was a fifty per cent solution of listerine, which also has the advantage of a very agreeable taste and odor.

Now it very often happens that the centers of decay about the teeth are filled with particles of food, and we do not in such cases have liquids to sterilize, but solid subtances impregnated with micro-organisms; what effect can we produce upon these by the action of the solutions given above?

To determine this question, a second series of experiments was made in the following manner:

Small porous bodies (bread, meat, paper, etc.), of as nearly the same size as possible, were saturated with solutions charged with certain micro-organisms, or with stale saliva, then subjected to the action of the antiseptic solutions during a specified length of time, and then put into culture gelatine and the number of colonies which developed determined. The stronger the antiseptic and the longer the time of exposure, the less will be the number of colonies which develop in the culture tube. As control, the experiment was repeated, using sterilized water instead of an antiseptic solution.

To avoid transferring too much of the antiseptic to the culture tube, the piece is placed for an instant on sterilized blotting paper, to remove the excess of liquid. I give the results of one of these experiments below. In this solution No. 5 was made use of, and *small* pieces of bread charged with bacteria subjected to the action of the solution 20, 35, 55, 70, 90 and 120 seconds respectively. The control tube developed 4,500 colonies:

| | | | | |
|---|---|---|---|---|
| Tube 1. | ( 20 seconds, action) | developed | 420 | colonies. |
| " 2. | ( 35 " " ) | " | 46 | " |
| " 3. | ( 55 " " ) | " | 250 | " |
| " 4. | ( 70 " " ) | " | 13 | " |
| " 5. | ( 90 " " ) | " | 1 | colony. |
| " 6. | (120 " " ) | " | remained sterile. | |

It may appear strange that tube 3 should develop more colonies than tube 2, but such irregularities often occur, owing to the fact that it is not possible to obtain pieces of bread or meat of *exactly* the same size and consistency. The result of the experiment is, however, very clear. When large compact pieces were used (as large as a pea, for example), such as may sometimes be found in cavities of decay, it required as much as ten to fifteen minutes to effect a complete sterilization. The lesson is plain. Even such a powerful wash as the one under consideration will accomplish but little in sterilizing the human mouth when the centers of decay are stuffed full of food. This is also the reason why excessive smoking, notwithstanding the fact that tobacco smoke is a powerful antiseptic, does not insure the teeth against decay; the smoke passes over the surface, but does not penetrate to the point of action. It follows that the use of the mouthwash should always be preceded by the thorough use of the brush or tooth-pick, removing at least all larger particles of food and opening the spaces between the teeth, so that the wash may penetrate to the vulnerable point. If this is conscientiously done, I am convinced that we have in solution No. 5, and, in a less degree, in the other solutions specified, a powerful means of preventing the excessive ravages of decay. The solutions, 2 and 5, may be made use of in the following form:

| | | |
|---|---|---|
| No. 2. | Thymol | 0.25 |
| | Benzoic acid | 3.00 |
| | Tinct. eucalypt | 15.00 |
| | Alcohol abs | 100.00 |
| | Oil of wintergreen | 25 drops. |
| | Or oil of peppermint | 20 " |

In use, enough of this mixture is added to a mouthful of water to produce a decided cloudiness.

The wash, no doubt, may be rendered softer and more palatable by the addition of glycerin, tinct. of katechu or something of the kind. Perhaps some one who is interested in mouthwashes will kindly undertake the task.

No. 5 is prepared in the same way, with the addition of 0.8 bichloride of mercury.

| | | |
|---|---|---|
| ℞. | Acid thymic | 0.25 |
| | —— benzoic | 3.00 |
| | Hydrarg. bichlorid | 0.80 |
| | Tinct. eucalypt | 15.00 |
| | Alcohol absolut | 100.00 |
| | Ol. gaultheria | gtt. xxv. |

Naturally, every one is shocked at the idea of putting bichloride of mercury in a mouthwash, but I think a more thorough consideration of the question will show that it is not so reprehensible an act as may at first appear.

The strength in which the bichloride is used in the mouth is about $\frac{1}{2000}$. Let us suppose that the patient swallows of the solution two grams daily (as a matter of fact, one need not swallow any at all); it would require one hundred days to have swallowed 0.1 grams of the salt, which is the maximum dose for one day. In this matter, however, reasoning is of little value; nowhere is the saying in medicine, experience is of greater value than reasoning, truer than in questions dealing with the physiological action of the salts of mercury. I, myself, have made extensive use of the above formula without a trace of any physiological or toxicological action, and if a sufficient number of members of the profession would make a trial of this solution upon themselves, and report the results in the INDEPENDENT PRACTITIONER, a great deal would be done towards solving the question of the advisability of recommending the wash in practice. The taste of the bichloride is disagreeable, even in very dilute solutions; it may, however, I hope, be disguised by the addition of proper substances.

Unfortunately, our pharmacopœia is not yet so rich that the physician or dentist can restrict himself to the use of good-tasting medicaments.

On the whole, after a very great number of experiments, I have come to the conclusion that a thoroughly efficacious mouthwash cannot be constructed with the substances now at command, without the use of bichloride of mercury.

